# Treatment outcomes and next-generation sequencing of a rare malignancy - urachal carcinoma: case report and literature review

**DOI:** 10.3389/fonc.2026.1744925

**Published:** 2026-04-21

**Authors:** Tian Tan, Xinhao Peng, Dan Shang, Linlin Zheng, Jie Chen, Hong Wu, Chenhui Cao, Senlin Xu, Chuan Xu

**Affiliations:** 1 School of Basic Medical Sciences, Chengdu University of Traditional Chinese Medicine, Chengdu, Sichuan, China; 2 Department of Oncology, Affiliated Hospital of Southwest Jiaotong University, The Third People’s Hospital of Chengdu, Chengdu, Sichuan, China; 3 Department of Oncology & Cancer Institute, Sichuan Academy of Medical Sciences, Sichuan Provincial People’s Hospital, School of Medicine, University of Electronic Science and Technology of China, Chengdu, Sichuan, China; 4 Jinfeng Laboratory, Chongqing, China; 5 Yu-Yue Pathology Scientific Research Center, Chongqing, China; 6 Integrative Cancer Center & Cancer Clinical Research Center, Sichuan Cancer Hospital & Institute, Sichuan Cancer Center, School of Medicine, University of Electronic Science and Technology of China, Chengdu, Sichuan, China; 7 Department of Pathology, Southwest Hospital, Army Medical University (Third Military Medical University), Chongqing, China

**Keywords:** chemoradiotherapy, FLT1, MYC, next-generation sequencing, targeted therapy, Urachal carcinoma

## Abstract

UrC is a rare malignancy with uncertain pathogenesis. The main symptoms include gross hematuria, abdominal pain, and an abdominal mass. The lack of comprehensive clinical analysis necessitates selecting an optimal therapeutic strategy for each patient. Here, we present a comprehensive review of the clinical manifestations, diagnosis, and treatment of UrC, illustrated with a case successfully managed through surgical intervention and adjuvant chemoradiotherapy. Meanwhile, the analysis of NGS detected two tumor-specific mutated genes: MYC (gene amplification, CN: 59.5) and FLT1 (missense mutation, c.1061G>A (p.R354Q), abundance: 2.1%). These findings may provide insights into tumor growth and guide therapeutic strategies.

## 1 Introduction

During intrauterine development, the bladder descends into the pelvis and the urachus connects the umbilical to the bladder. Gradually, the urachus degenerates and forms a fibromuscular canal referred to as the median urachal ligament (1). In about one-third of adults, urachus residues still can be found, which makes the occurrence of UrC possible (2). UrC is rare in adults, with a significantly higher incidence in males. The patients are almost over 50 years old, and the pathological types are mucoid adenocarcinoma (69%), non-mucoid adenocarcinoma (15%), sarcoma (8%), transitional cell carcinoma (3%), squamous cell carcinoma (3%) and others (2%) (3). The secluded location and lack of early symptoms make UrC difficult to be detected and prone to recurrence and distant metastasis. In previous case reports, surgery was the main treatment method, and chemotherapy, radiotherapy or targeted therapy could be given selectively (4, 5). With the development of NGS, more tumor-related genetic mutations have been found and provide a high-resolution and global view of the cancer genome. However, data on clinical outcomes and genomic profiles in UrC are scarce due to its low incidence. Consequently, an adequate sample size is required to establish patterns in disease pathogenesis and therapeutic outcomes for future studies.

Here, we report a case of young female patient diagnosed with urachal mucinous cystadenocarcinoma. Following multimodal therapy, the patient remained disease-free with no evidence of recurrence or metastasis during four years of scheduled follow-up. Moreover, NGS analysis identified multiple gene mutations. These findings suggest implications for the pathogenesis and treatment of urachal mucinous cystadenocarcinoma. In conjunction with the present case, a comprehensive review of the literature related to UrC is provided. This review summarizes the clinical features and therapeutic approaches for UrC, and further analyzes the genetic alterations identified via NGS, providing a reference framework for managing this disease.

## 2 Case presentation

**Figure 1:**
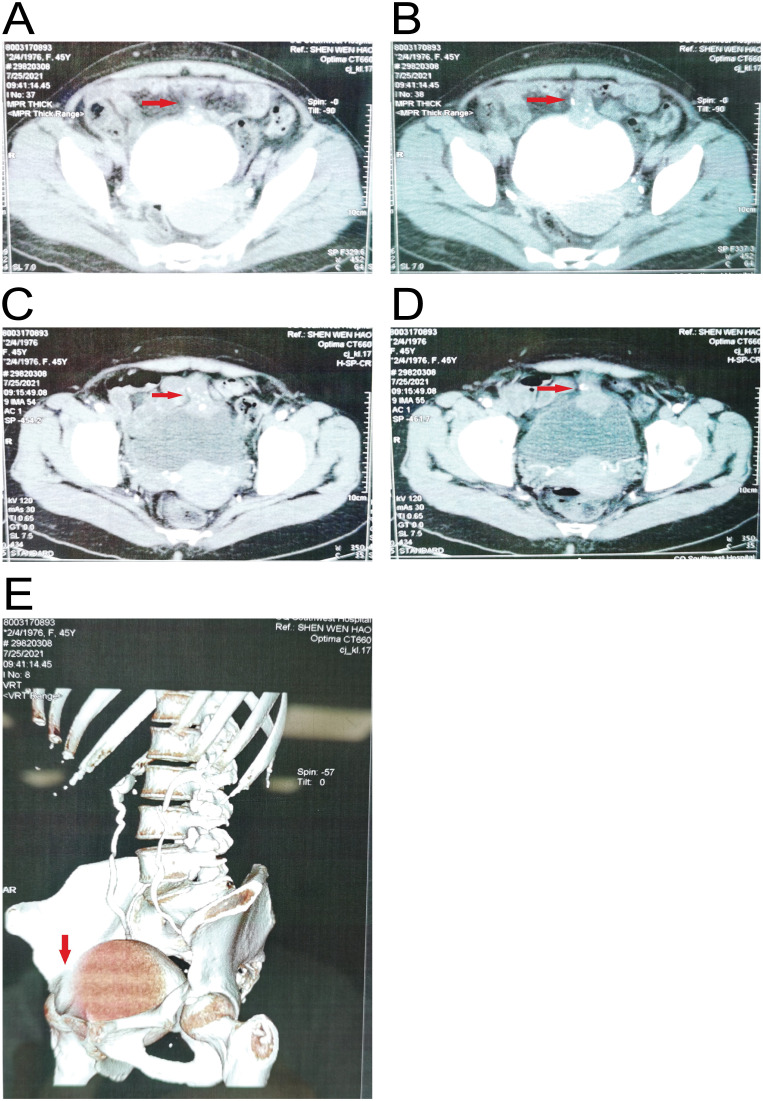
Images of the patient before operation. **(A, B)** The anterior wall of the bladder exhibits irregular soft tissue density, and the tumor extends into and out of the cavity. Calcifications are seen in the tumor area, and the maximum cross-sectional area of the tumor is about 4.9*3.1cm; **(C, D)** Enhanced CT shows mild inhomogeneous enhancement. The excretory stage shows a filling defect, and there is no obvious enlarged lymph node shadow around. **(E)** CT three-dimensional imaging of the ureter shows a space-occupying lesion in the anterior wall of the bladder.

**Figure 2:**
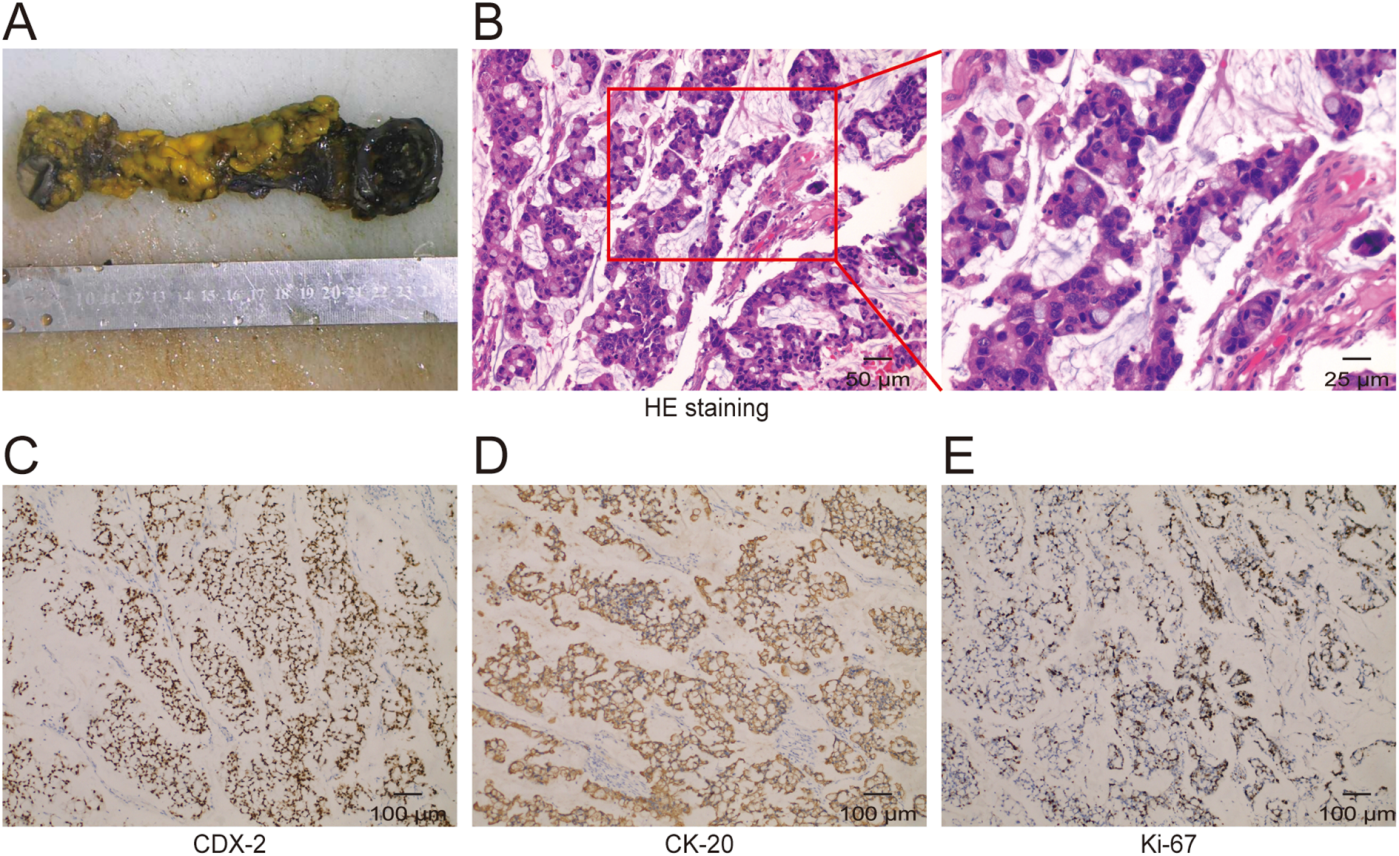
Tissue and pathology. **(A)** Gross pathology showing the resected urochal carcinoma specimen. **(B)** HE staining of the surgical specimen. **(C-E)** Immunohistochemical results of the surgical specimen.

A 45-year-old woman with no significant previous medical history was sent to Southwest Hospital Urology Department in July 2021 due to recurrent painless gross hematuria. Both ultrasound and MRI revealed the presence of tumor mass in the anterior wall of the bladder. Besides, CT ureteral imaging also suggested the presence of a mass on the anterior wall of the bladder extending toward the umbilical (**Figures 1A–D**). Three-dimensional CT imaging also showed a clear soft tissue density shadow in the anterior wall of the bladder (**Figure 1E**), which increased the likelihood of UrC. She underwent robotic-assisted partial bladder and umbilical resection and bilateral pelvic lymph node dissection in July 2021. The surgically removed parts include part of the bladder, urachal, tumor, and umbilicus, and the lump size is about 3*2.9*2cm (**Figure 2A**). Postoperative pathological examination revealed urachal mucinous cystadenocarcinoma with tumor involvement extending through the full thickness of the adjacent bladder wall. Meanwhile, lymph node metastases were found on each side of the pelvic cavity. Hematoxylin-eosin (HE) staining indicated malignancy, consistent with mucinous cystadenocarcinoma of urachal origin (**Figure 2B**). Immuno-histochemistry (IHC) staining detected the positive expression of CDX-2, CK20, and Ki-67 (**Figures 2C–E**).

**Figure 3:**
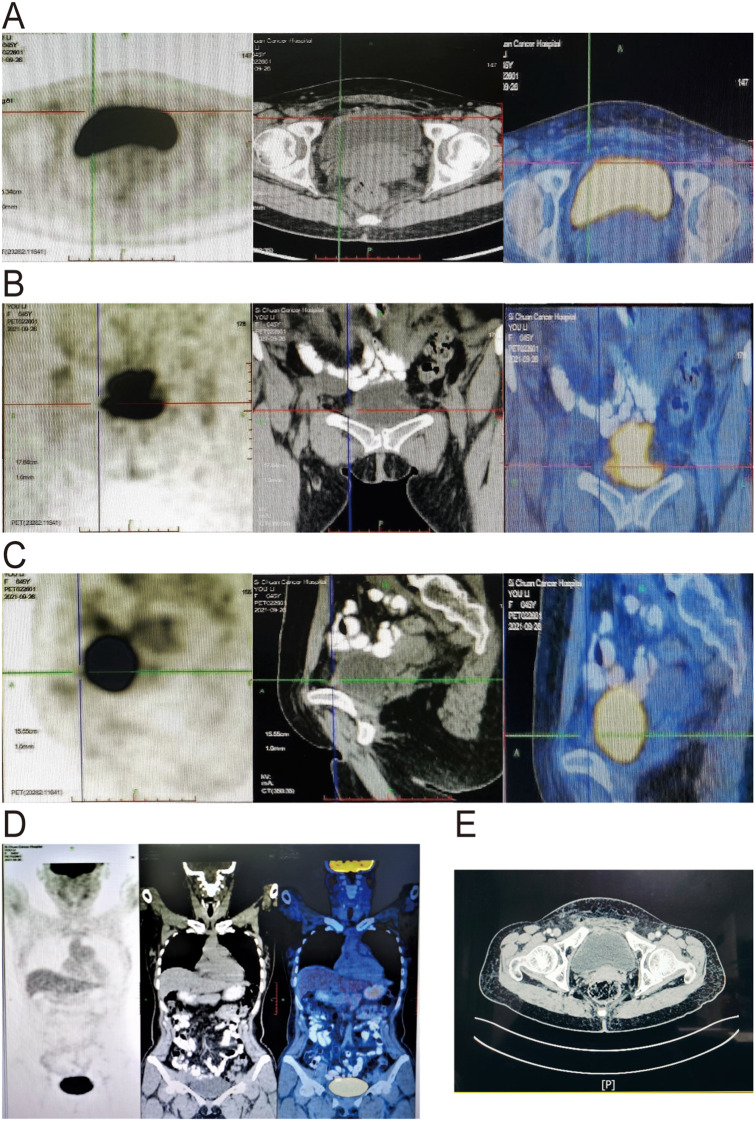
Follow-up images. **(A-D)** PET-CT showed postoperative changes with a slightly thickened anterior bladder wall predominantly on the right side, without significant metabolism. **(E)** CT showed postoperative changes with a slight thickening of the anterior bladder wall.

The NGS (Burning Stone Biotechnology, Guangzhou, China) was performed and the sequencing results identified two tumor-specific mutation genes: MYC (gene amplification, CN:59.5) and FLT1 [missense mutation, c.1061G>A (p.R354Q), abundance: 2.1%], but microsatellite instability index and other immune-related factors were not detected. Other mutations related to drug metabolism are shown in **Table 1**. For further treatment, this patient underwent two cycles of oxaliplatin and capecitabine chemotherapy at the original hospital in August 2021. Next, this patient was transferred to Sichuan Cancer Hospital in September 2021 and underwent PET-CT which showed that there was no distant metastasis (**Figures 3A–D**). Due to the positive pathological margin, the patient is receiving concurrent chemoradiotherapy. The chemotherapy regimen was gemcitabine + capecitabine. And radiation therapy regimens are as follows: the prescribed doses were 62.5 Gy to the positive lymph nodes of the gross tumor volume (GTVn), 62.5 Gy to the tumor bed clinical target volume (CTVtb), and 55 Gy to the high-risk clinical target volume (CTV1), delivered in 25 fractions. The irradiation was administered once daily, 5 days a week. Subsequently, the patient underwent two cycles of oxaliplatin and capecitabine chemotherapy. This patient underwent rigorous postoperative monitoring, including physical examinations, laboratory tests, and enhanced CT scans of the abdominal and pelvic cavity every 3 to 6 months. At the latest follow-up of 48 months, the patient was alive, and no significant treatment-related delayed toxicities were observed. And CT scan result showed no recurrence or metastasis, and the bladder showed postoperative changes (**Figure 3E**).

**Table 1:** Polymorphism of enzymes associated with drug metabolism from NGS.

Gene	Variation	Mutant	Prediction (chemotherapy effect)
GSTM1	Homozygous deletion polymorphism	–	Better than wild type GSTM1 (Platinum)
GSTP1	p.I105V (Heterozygous polymorphism)	c.313A>G (p.I105V)	Better than wild type GSTP1 (Platinum and Anthracycline)
MTHFR	p.A222V (Homozygous polymorphism)	c.665C>T (p.A222V)	Better than wild type MTHFR (5-Fu); Toxic effect (Methotrexate)
NQO1	p.R139W (Heterozygous polymorphism)	c.415C>T (p.R139W)	Worse than wild type NQO1 (Mitomycin)
UGT1A1	p.G71R (Heterozygous polymorphism)	c.211G>A (p.G71R)	Toxic effect (Irinotecan and Etoposide)
XRCC1	p.Q399R (Homozygous polymorphism)	c.1196A>G (p.Q399R)	Better than wild type XRCC1 (Platinum)

## 3 Minireview of literature

As a rare malignant tumor, comprehensive understanding of UrC remains limited. The clinical manifestations of UrC are relatively hidden, typically presenting with urinary system symptoms, and are difficult to distinguish from bladder urothelial malignancies. While surgery serves as the primary treatment option, the evidence supporting therapeutic interventions for metastatic or advanced-stage disease is limited and inconsistent. Therefore, tailored treatment plans must be implemented based on individual patient characteristics. In addition, with the widespread adoption of genomic sequencing technologies, an increasing number of UrC-associated therapeutic targets have been identified. Thus, targeted therapy, immunotherapy, and other approaches might be crucial in the future treatment of UrC.

### 3.1 Clinical manifestations and diagnosis

For UrC, male patients are more common than females, and the common clinical symptoms include gross hematuria, abdominal pain, abdominal mass, dysuria, and other systemic symptoms such as nausea, fever, weight loss, or diarrhea (4). Based on the existing case analysis, cystoscopy plays an important role in the diagnosis of disease, and imaging tests such as computer tomography (CT), 18F-FDG PET CT, magnetic resonance imaging (MRI), and ultrasound are also essential (6–8). It has been reported that most UrCs are adenocarcinomas and have the same embryonic origin as the colon. Therefore, some gastrointestinal tumor markers can also be used to monitor the disease progression of UrC such as CEA, CA125, CA19-9, CA15-3, and alpha-fetoprotein. The most sensitive of them are CA19–9 and CEA (9, 10).

### 3.2 Tumor staging and prognosis

Currently, Sheldon and Mayo are the most common UrC staging systems (11, 12). The TNM staging of bladder cancer is not suitable for UrC (13). Recently, some scholars proposed a new TNM staging system that has a more balanced sample distribution and accurate correlation between staging and survival (14). Detailed information on various staging systems is shown in **Table 2**. Some epidemiological studies show significantly different survival between stage IIIA (-) and stage IIIB (+) disease (according to the Sheldon staging system) (15). Meanwhile, a retrospective analysis indicated that patients with positive surgical margins have a 60% lower 5-year survival rate than those with negative margins (16), suggesting that margin status may be an independent prognostic factor. In addition, lymph node or distant metastasis also influences the treatment and prognosis of UrC (6).

**Table 2:** Various staging systems for urachal carcinoma.

Sheldon (11)	Mayo (12)	Ontario (42)	New TNM staging (14)
I: no invasion beyond urachal mucosa	I: confined to the urachus and/or bladder	I: confined to the submucosa	I: Limited to the urachal submucosa
II: invasion confined to urachus	II: extending beyond the muscularis of the urachal and/or bladder	II: confined to the muscular wall of the bladder	II: invasion of bladder muscularis propria or microscopic invasion of bladder perivesical tissue
III: extending to A: bladder B: abdominal wall C: peritoneum D: organs other than the bladder	III: infiltrating regional lymph nodes	III: extending into the peri-urachal or vesical soft tissue	III: macroscopic invasion of bladder perivesical tissue or invasion of adjacent tissues, including the uterus, vagina, and prostate
IV: metastasis to A: lymph nodes B: distant parts	IV: infiltrating of non-regional lymph nodes or distant metastasis	IV: invasion of adjacent organs including the abdominal wall	IV: invasion of any nodal or distant location, or of the pelvic, abdominal wall, or peritoneum

### 3.3 Surgery and chemoradiotherapy

According to the NCCN Guidelines (Bladder Cancer Version 1. 2025), the recommended treatment for UrC is surgery or radiotherapy to achieve local control, combined with best supportive care. Current surgical approaches encompass extended partial cystectomy with en-bloc resection of the urachal mass, urachal remnant, and umbilicus, combined with pelvic lymph node dissection. For larger masses, radical cystectomy is recommended (7, 12). In fact, the guidelines indicate no current evidence supports the use of neoadjuvant or adjuvant chemotherapy in patients with pure adenocarcinoma of the bladder, including UrC. However, the following suggestions are given for systemic treatment strategies. For patients with node-positive pure adenocarcinoma, guidelines have consistently recommended the FOLFOX regimen (oxaliplatin, leucovorin, and fluorouracil) and the GemFLP regimen (fluorouracil, leucovorin, gemcitabine, and cisplatin) (17). For the metastatic, and advanced disease, consider using the NCCN Guidelines for Colon Cancer (https://www.nccn.org/professionals/physician_gls/pdf/bladder.pdf). Analysis of colon cancer adjuvant therapy guidelines reveals that CAPEOX (oxaliplatin and capecitabine) or FOLFOX regimens demonstrate superior efficacy over 5-FU/leucovorin in stage III colon cancer patients (18). Furthermore, the 3-month CAPEOX regimen maintains comparable efficacy while significantly reducing toxicity (19, 20).

This patient is classified as either Sheldon stage IVA or Mayo stage III. According to the guidelines and clinical case discussion, firstly, we surgically resected part of the bladder, urachal, tumor, and umbilicus. Subsequently, we administered oxaliplatin and capecitabine (CAPEOX regimen) chemotherapy to the patient, which enhances therapeutic efficacy and reduces toxicity. The drug metabolism information in **Table 1** also suggests that certain gene mutations (GSTM1, GSTP1, and XRCC1) are associated with better responses to platinum. To reduce the risk of recurrence and metastasis associated with positive surgical margins and lymph node positivity, the patient underwent radiotherapy with adjuvant treatment of gemcitabine and capecitabine. After a four-year follow-up, the patient has shown no recurrence or metastasis. Previously, a meta-analysis of 1010 cases showed that, in the absence of effective radiotherapy, chemotherapy is the only treatment option with the potential to prolong survival (4). However, our case provides a useful reference for the radiotherapy management of UrC. Furthermore, some previous case reports have shown that 5-FU regimens are more effective than cisplatin-based chemotherapy regimens, and that the combination of antimetabolites and platinum chemotherapy drugs can produce the strongest anti-tumor effects. However, in the absence of prospective clinical trial data, treatment decisions must be individualized. Based on a comprehensive analysis of this clinical case, the patient presented with advanced tumor stage and positive surgical margins. Consequently, concurrent chemoradiotherapy was considered likely to significantly reduce the risk of recurrence and metastasis. Furthermore, the follow-up data to date demonstrate the efficacy of this regimen, with no evidence of recurrence or metastasis observed.

### 3.4 Targeted therapy based on genomic sequencing

Given the limited efficacy of chemotherapy for UrC, recent research has yielded important breakthroughs in exploring targeted therapeutic approaches. Genomic profiling studies have identified high-frequency driver mutations in UrC, including TP53 (70%), KRAS (28.3%), MYC (20.3%), SMAD4 (18.2%), and GNAS (18%) (21). Furthermore, studies have found that mutations in targetable genes such as EGFR, HER2, and BRCA are also present in UrC (21, 22). Inhibitors of related targets (such as EGFR inhibitors, PARP inhibitors, and MEK inhibitors) and immune checkpoint inhibitors have demonstrated preliminary clinical efficacy in case reports. For instance, responses to anti-EGFR therapy have been observed in patients harboring EGFR amplifications (4, 23). And another UrC patient with MSH6 mutation achieved stable disease after anti-PD-L1 therapy (23, 24). Recent investigations have revealed a predominantly immunosuppressive tumor microenvironment (TME) in UrC, characterized by an abundance of immunosuppressive immune cells, which provides a compelling rationale for PD-1/PD-L1 checkpoint blockade (25, 26).

In this case, NGS revealed a high copy number amplification of MYC (CN: 59.5). Additionally, a missense mutation in FLT1 (p.R354Q) was detected, although at a low variant allele frequency (VAF) of 2.1%. In bladder cancer patients, alterations in the MYC gene were observed in 7.06% of cases, with amplification (6.33%) and mutation (0.73%) being the most prevalent subtypes. In addition, alterations in the FLT1 gene were identified in 3.16% of cases, predominantly consisting of mutation (1.95%) and amplification (1.22%) [the information was obtained from cBioPortal (www.cbioportal.org)]. With additional NGS results from more cases, the molecular landscape of UrC can be further enriched. Ultimately contributing to the elucidation of its etiology and guiding clinical medication (27). These two genes are summarized as follows.

#### 3.4.1 MYC

For MYC, the gene was altered in 6.57% of 594 cases of colorectal adenocarcinoma and 7.06% of 411 cases of bladder urothelial carcinoma. More than 50% of human malignancies have dysregulated MYC family oncogenes, which are linked to a poor prognosis and a low survival rate (28). Myc is involved in multiple processes of tumor progression, such as proliferation, apoptosis, differentiation, and metabolism. Additionally, MYC can increase immunosuppressive cytokines like TGFβ and decrease the production of antigen-presenting proteins like MHC I, which prevents cytotoxic T cells from acting and encourages immune evasion (29–31). Therapeutic drugs directly targeting MYC are under development, and their clinical efficacy remains to be verified (29). Encouragingly, scientists from Spain have developed a new therapy (Omomyc) targeting MYC-MAX heterodimer to inhibit MYC gene, which is most promising for clinical application (29, 32). Other promising candidates include inhibitors of the PI3K–AKT–mTOR pathway, inhibitors of the translation initiator eIF4A (silvestrol), PIN1 inhibitors, antisense oligonucleotides, etc. (33, 34). In contrast to colorectal and bladder tumors, MYC is markedly elevated in UrC and might be a special indicator of UrC (35).

#### 3.4.2 FLT1

For FLT1 (fms-like tyrosine kinase), the gene was altered in 9.26% of 594 cases of colorectal adenocarcinoma and 3.16% of 411 cases of bladder urothelial carcinoma. Although the FLT1 (p.R354Q) mutation was detected in this patient, the VAF was only 2.1%, suggesting that the FLT1 mutation is likely subclonal and present in only a minority of tumor cells. Nevertheless, this finding underscores the intratumoral heterogeneity characteristic of complex solid tumors.

This gene encodes tyrosine kinase receptors which have been identified as high-affinity VEGF receptors (36). The VEGFR family consists of three receptors, namely VEGFR1 (FLT1), VEGFR2 (KDR/FLK1), and VEGFR3 (FLT4) (37). The role of FLK1 and VEGFA in tumor angiogenesis has been fully confirmed, however, the signal transmission related to FLT1 is still controversial. Currently, research has revealed that FLT1 is highly associated with embryonic vasculogenesis, macrophage function, and the pre-metastatic niche. Meanwhile, FLT1 inhibits angiogenesis in endothelial cells due to inadequate tyrosine phosphorylation (38, 39). NGS identified a missense variant (p.R354Q) in the FLT1 gene, resulting in an amino acid substitution within the I-SET domain. The I-SET domain, an immunoglobulin-like structural motif, is conserved across diverse protein families, including receptor tyrosine kinases, hemolin, titin, telokin, twitchin, and axonin-1 (40). This domain plays crucial roles in mediating cellular recognition, receptor interactions, muscle architecture, and immune system function (41). A retrospective study suggests that targeting FLT1 and PlGF can inhibit tumor growth. Currently available drugs targeting FLT1 include cabozantinib, cediranib, midostaurin, etc., but their clinical effectiveness needs further confirmation (37). The missense variant of this gene may render FLT1-targeted drugs ineffective.

Although the current evidence for targeted and immunotherapeutic interventions in UrC remains limited to isolated cases, these collective findings herald the advent of the era of precision medicine in UrC management. Unfortunately, no suitable targeted drugs are currently available for this case.

## 4 Conclusions

UrC is a rare tumor with a poor prognosis. Currently, surgery is the preferred treatment method, and chemotherapy and radiotherapy can stabilize the condition to a certain extent. However, due to a lack of extensive clinical analysis, the treatment of this disease remains to be further explored. For different patients, reasonable chemoradiotherapy regimens may reduce the risk of postoperative recurrence and metastasis. This patient underwent a multimodal treatment approach involving surgery, adjuvant chemotherapy, and concurrent chemoradiotherapy, which resulted in a four-year disease-free survival. This favorable outcome suggests that this regimen may achieve durable disease control with manageable toxicity for this rare urachal malignancy. In addition, mutational analysis of individual targets and diverse pathways suggests the promising potential of targeted therapy as a future alternative. Therefore, future investigations should focus on characterizing the molecular features of UrC, investigating its oncogenic processes, and improving clinical decision-making.

## Data Availability

The original contributions presented in the study are included in the article/supplementary material, further inquiries can be directed to the corresponding author/s.
