# Supplementary figures and images for: Treatment outcomes and next-generation sequencing of a rare malignancy - urachal carcinoma: case report and literature review
Source: Front Oncol. 2026 Apr 21;16:1744925. doi: 10.3389/fonc.2026.1744925 (PMC13138954; doi:10.3389/fonc.2026.1744925)

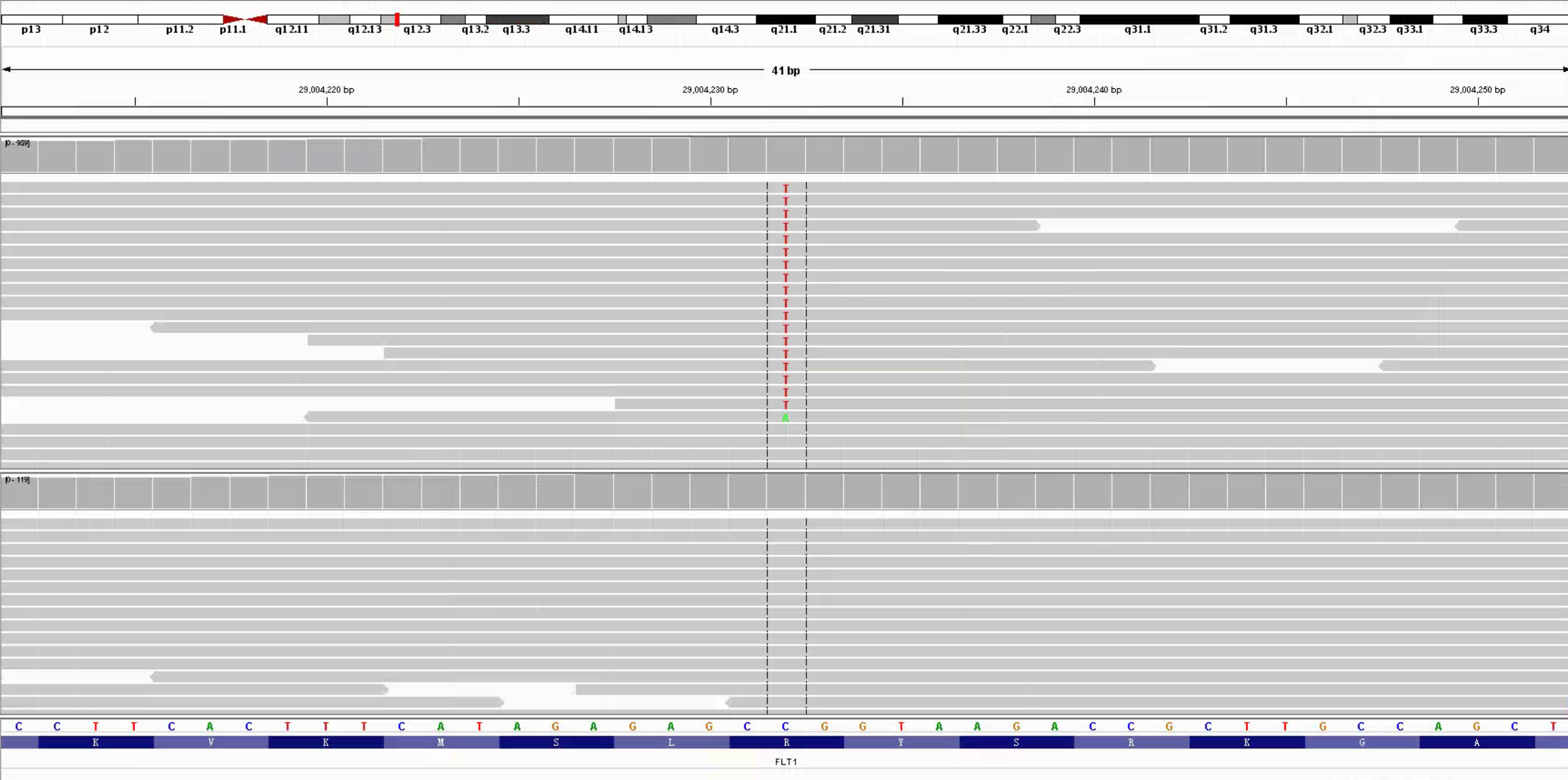

Supplement: Supplementary file 1 [file SupplementaryFile1.png]

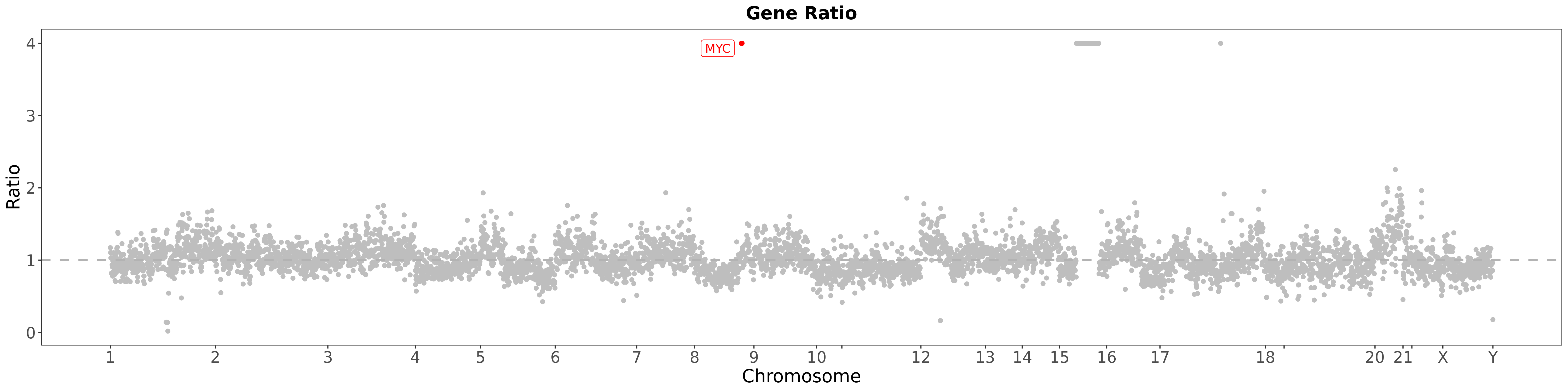

Supplement: Supplementary file 2 [file SupplementaryFile2.png]
